# Ice-core evidence of earliest extensive copper metallurgy in the Andes 2700 years ago

**DOI:** 10.1038/srep41855

**Published:** 2017-01-31

**Authors:** A. Eichler, G. Gramlich, T. Kellerhals, L. Tobler, Th. Rehren, M. Schwikowski

**Affiliations:** 1 Paul Scherrer Institut, CH-5232 Villigen PSI, Switzerland; 2 Oeschger Centre for Climate Change Research, University of Bern, CH-3012 Bern, Switzerland; 3 Department for Chemistry and Biochemistry, University of Bern, Freiestrasse 3, CH-3012 Bern, Switzerland; 4 UCL Institute of Archaeology, 31-34 Gordon Square, London WC1H 0PY, UK; 5 College for Humanities and Social Sciences, HBKU Doha, Qatar

## Abstract

The importance of metallurgy for social and economic development is indisputable. Although copper (Cu) was essential for the wealth of pre- and post-colonial societies in the Andes, the onset of extensive Cu metallurgy in South America is still debated. Comprehensive archaeological findings point to first sophisticated Cu metallurgy during the Moche culture ~200–800 AD, whereas peat-bog records from southern South America suggest earliest pollution potentially from Cu smelting as far back as ~2000 BC. Here we present a 6500-years Cu emission history for the Andean Altiplano, based on ice-core records from Illimani glacier in Bolivia, providing the first complete history of large-scale Cu smelting activities in South America. We find earliest anthropogenic Cu pollution during the Early Horizon period ~700–50 BC, and attribute the onset of intensified Cu smelting in South America to the activities of the central Andean Chiripa and Chavin cultures ~2700 years ago. This study provides for the first time substantial evidence for extensive Cu metallurgy already during these early cultures.

Andean cultures developed one of the great metallurgical traditions of the ancient world, but they have been far less investigated and understood than the ones in the Middle East and Europe, or Asia. For Andean metallurgy copper (Cu) in particular was an important resource and still plays a central economic role in many South American countries today. All ancient Andean alloys, except the naturally occurring gold-silver (Au-Ag) alloy, contained Cu. This metal is therefore often referred to as the “backbone of Andean metallurgy – the mother of all Andean metals”^1^. Despite of this importance the onset of extensive Cu metallurgy in South America is still debated.

Most direct evidence is provided by archaeological artefacts. Earliest Cu artefacts in the central Andes found at Mina Perdida in Peru date back to ~1410 to 1090 BC^2^. They consist of native Cu and Au hammered into thin foils, documenting that working of native Cu preceded the production of smelted Cu objects^2^. A first hint of the use of molten Cu is a Cu-Ag bead from the Peruvian site of Malpaso dated to ~1000 BC^3^,^4^. Thus, temperatures of about 1000 °C were achievable at that time and it is very likely that South American Cu production by smelting started soon after 1000 BC. The verification of this hypothesis is challenging since very few extant artefacts are known from that early period and ancient metallurgical sites are difficult to find because of the lack of substantive remains, particularly smelting installations. Prehistoric smelting furnaces tended to be small or smelting was performed on open fires and thus left little permanent remains^5^.

The scarceness of Cu artefacts holds true also for the following ~1000 years. Among the archaeological findings attributed to the Chavin culture in Peru (~900–200 BC) most were produced from native Au or Au-Cu-Ag alloys and only very few from pure Cu^6^,^7^,^8^,^9^. Further south, Cu slag was found at Wankarani on the Bolivian Altiplano and placed in a stratigraphic context of ~850–650 BC^10^,^11^, but the metallurgical nature of these slags is questioned and controversially, a volcanic origin was proposed^12^. At excavations of Chiripa sites close to the Lake Titicaca human burials were found, frequently accompanied by wealth items including few artefacts of Cu and Au dated to ~800–400 BC (Christine Hastorf, personal communication 2016^13^). In Northern Chile, at the Ramaditas site, earliest traces of Cu metallurgy were detected from the first centuries BC^11^. Finally, the first strong evidence for highly sophisticated Cu smelting came from the rich Cu artefacts from the Moche culture in northern Peru (200–800 AD)^4^,^14^.

Complementary to archaeological artefacts, natural archives such as ice cores, peat bogs or lake sediment cores contain independent information about the smelting history by recording a metallurgy-related air pollution signal. To our knowledge the only data set covering the entire time period of potential Cu metallurgy is a 4200-year peat bog Cu record from Tierra del Fuego, an archipelago at South America’s southernmost tip, roughly 3,000 km south of the major Cu mining activity centers^15^. The record suggested that earliest anthropogenic air pollution potentially originating from Cu metallurgy occurred already around ~2000 BC, thereby considerably preceding any archaeological evidence. Two other records are much shorter, covering only the last ~1400 years, and do therefore most likely not contain the signals of earliest Cu metallurgy. The ice core from the Quelccaya ice cap in Peru revealed a widespread anthropogenic signal from Cu metallurgy after about 1540 AD, corresponding to the beginning of colonial mining and metallurgy in Peru and Bolivia^16^. A sediment core record from Lake Pirhuacocha in the central Peruvian Andes provided evidence for earliest extensive Cu smelting around 1000 AD, coinciding with the fall of the Wari Empire and decentralization of local populations^17^.

In this study we present a 6500-year history of Cu emissions in the central Andes based on a continuous, highly time-resolved ice core record from the Illimani glacier, located at the north-eastern margin of the Bolivian Altiplano^18^. Potential source regions of air pollution arriving at the Illimani include main parts of Peru, Bolivia, northern Chile, and north-western Brazil as shown by the 5-day back-trajectory analysis (Fig. 1), thus encompassing the cradle of New World metallurgy in the Central Andes^19^,^20^,^21^. The ice core archive has already been proven to record the history of Andean pre- and post-colonial Ag metallurgy during the past 2000 years^21^. The Cu record allowed us to constrain the earliest stages of extensive prehistoric Cu metallurgy in South America and to document the complete history, including timing and intensity of Cu smelting activities.

## Results and Discussion

### Differentiation between natural and anthropogenic sources of Cu

The most pronounced maxima in the Illimani Cu concentration record occur in 4500–2800 BC and 700–200 BC, followed by generally increased levels from ~500 AD and a sharp spike from 1900–2000 AD (Fig. 2). This signature combines two sources of atmospheric Cu, namely natural emissions of mineral dust and the imprint from mining and metallurgical processing, which need to be disentangled for interpreting the anthropogenic signal. An input from natural dust is indicated by a simultaneous concentration increase in lithogenic elements such as Ce. The close correlation between ice core Cu and Ce concentrations from 4500 to 2800 BC suggests that the earliest Cu maximum is related to increased deposition of mineral dust. High mineral dust concentrations in the ice core are generally a sign for droughts and in this case coincide with lowest water levels of the nearby Lake Titicaca of the past 25,000 years^22^,^23^ (Fig. 2).

To discriminate between the natural and anthropogenic origin of Cu, enrichment factors (EFs) were calculated. EFs are ratios of trace element concentrations to a lithogenic element such as Al, Ce, Fe, La, Nd, and Ti, which are normalized to the same elemental ratios of a reference material such as the global upper continental crust (UCC) or the regional background. We used Ce as lithogenic element^21^ following the conventional equation:


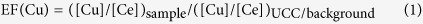


Average values of the UCC are not necessarily representative of the regional mineral dust composition, due to the fact that fractionation processes can occur during weathering, mobilization, and atmospheric transport. In this study we took advantage of the long record extending far back in time to determine a site-specific natural background Cu/Ce ratio for the regional dust from the ice core section 4500–2000 BC for which anthropogenic influence is assumed to be negligible. The ice core based background [Cu]/[Ce] ratio of 0.87 is higher compared to the UCC ratio of 0.22^24^, suggesting that the mineral dust composition in the study region is not represented by mean UCC values. The EF calculation was repeated using La and Nd as lithogenic elements (see Supplementary Fig. S1). The different Cu EFs are in remarkable good agreement, indicating that the record of the Cu EFs is not dependent on the chosen lithogenic element.

Contrary to the Cu concentrations, Cu EFs are low during the dry period 4500–2800 BC (Figs 2 and 3), confirming the underlying assumption of negligible anthropogenic input and indicating that the contribution of mineral dust was successfully removed by calculating EFs. A first minor increase of the Cu EFs occurred ~3000 BC (Fig. 3). This time is within the horizon of the Pre-ceramic period in the Central Andes, when Neolithic cultures dominated and no traces of any metal working are known. We therefore excluded a contribution of anthropogenic emissions and interpreted the fluctuations of the Cu EFs during the period 4500–2000 BC as variability of the regional mineral dust composition. Mineral deposits in the Central Andes consist of sedimentary rocks and polymetallic veins containing different Cu minerals as malachite, cuprite, chalcocite, bornite, chalcopyrite^25^. The composition of the deposits strongly varies between different regions (e.g. sulfide vs. oxide ores), but also within one ore body there is a strong inhomogeneity. This is the reason that there is not a constant Cu EF for the period 4500–2000 BC, but background fluctuations due to the varying Cu/Ce ratios in different local minerals. To account for varying background composition, only Cu EFs exceeding for at least a 100 years period (two consecutive data points) the mean +2σ level of the period 4500–2000 BC (EF = 1.6) were considered to be indicative of anthropogenic Cu pollution from extensive metallurgical processing (Fig. 4). Accordingly, pronounced maxima in the Cu EFs occur during ~700–50 BC (mean ± s.e. = 2.3 ± 0.2), 500–850 AD (2.3 ± 0.3), 1400–1500 AD (1.7 ± 0.1), 1600–1750 AD (2.1 ± 0.05) and in the 20^th^ century (9.7 ± 6.7). Cu EFs were clearly highest in the second half of the 20^th^ century (16.4), which is expected due to the large scale Cu production. Noteworthy are the high levels of enrichment already present more than 2000 years ago, outpacing even those during the Colonial era. In the following section we discuss these maxima in anthropogenic Cu emissions in relation to archaeological findings from the dominant civilizations during the pre-Columbian cultural periods (Initial, ~2000–1000 BC; Early Horizon ~1000 BC–200 AD; Early Intermediate, ~200–600 AD; Middle Horizon, ~600–1000 AD; Late Intermediate, ~1000–1450 AD; Late Horizon, 1450–1532 AD), Colonial times (1532–1900 AD), and the industrial era (20^th^ century).

### Earliest anthropogenic Cu maximum (~700–50 BC) - Chiripa/Chavin cultures

**Figure 1:**
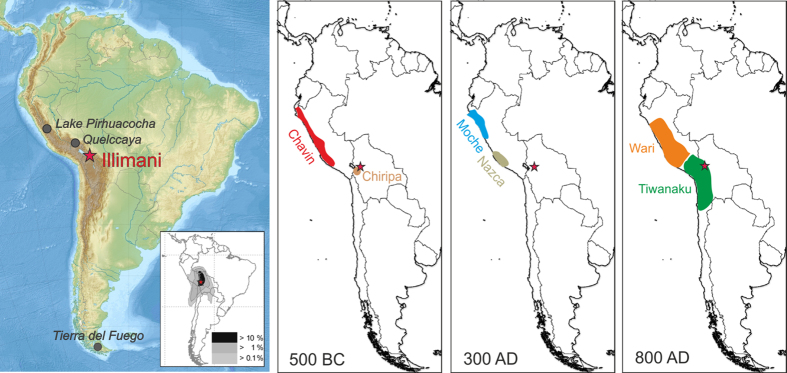
Location of the Illimani and sites mentioned in the text together with the earliest cultures in South America. *Left*: Map of South America (Credit Uwe Dedering, CC BY-SA 3.0, http://creativecommons.org/licenses/by-sa/3.0 or GFDL, http://www.gnu.org/copyleft/fdl.html, via Wikimedia Commons (2010) without changes, Date of access: 31/05/2016) with the Illimani (red star), the inset shows the frequency plot of 5-day back trajectories for the Zongo valley close to the Illimani for the period 1989–1998 using HYSPLIT and the NCEP/NCAR reanalysis. Back trajectories were run every 6 hours. *Right*: The schematic extension of the earliest cultures in South America around 500 BC, 300 AD, and 800 AD.

The earliest maximum of the Illimani Cu record related to emissions from Cu metallurgy occurs during the period ~700–50 BC (Fig. 4). This peak in anthropogenic Cu emissions coincides with the timing of the largest extent of the first complex societies around the Lake Titicaca (Chiripa culture) and the development of the first civilization in the Peruvian Andes (Chavin culture) (see Fig. 1). Although the Early Horizon Chiripa culture was among the most highly developed cultures in the region^26^, there is no indication of metallurgical activities until the Late Chiripa phase (800–200 BC), marked by the formation of a chiefdom^27^. Cu artefacts including beads and pins (see Fig. 5) have been excavated at Late Chiripa sites^13^,^27^. Early Horizon Cu bracelets were found in the Cochabamba valley^28^. In parallel with the Late Chiripa the Chavin culture developed in the northern Andean highlands of Peru from 900 to 200 BC. Under Chavin influence, a rapid development in metallurgical technology and an increasing use of metal objects occurred. Artefacts including crowns, diadems, nose rings, ear pendants, pins, spoons, necklaces, and tweezers have been found at tombs in the valley of Lambayeque, the north Sierra and Kuntur Wasi in Cajamarca^29^. However, the majority of the artefacts were made of Au or a ternary Au-Ag-Cu alloy^14^ with up to 20% Cu^6^, and only a few pure Cu artefacts have been reported^7^,^8^. Overall the findings originating from the Late Chiripa and Chavin period seem insufficient to explain the magnitude and extent of the observed ice core Cu EF maximum during the late first millennium BC. We assume that this maximum was caused by initial simple Cu metallurgy, which consisted of melting ores such as malachite in open fires. Such a technique is not very efficient for Cu production, but effective in emitting a relative large amount of Cu compared to the furnaces of the later Moche and Sicán civilization (see below). This would explain the discrepancy between limited archaeological evidence and the ice core data, and attribute the onset of intensified Cu smelting in South America to the activities of the central Andean Chiripa and Chavin cultures about 2700 years ago.

### Cu enrichment in the first millennium AD - Nazca/Moche and Tiwanaku/Wari

With the beginning of the first millennium AD anthropogenic Cu decreased to background levels (Fig. 4), most likely as a consequence of the decline of the Chavin and Chiripa civilizations. Around the same time the Nazca culture centred in southern coastal Peru emerged (~100 BC–650 AD) (Fig. 1). In contrast to the northern coast of Peru and the central Andean Altiplano, southern Peru was never a centre of metallurgical inventions or innovations^30^. Accordingly, during the Nazca period metallurgy principally remained on a basic level of cold working of Au and simple Cu working techniques, as indicated by Cu beads found at a Middle Nazca tomb at La Muña (400 AD)^30^. In agreement with that Cu EFs remained at background values during the Nazca period, supporting the archaeological evidence for limited extractive metallurgy.

During the first millennium AD ice core Cu EFs exceed the background range in the period 500–850 AD, reaching comparable levels to the earliest maximum (Fig. 4). In parallel with the Nazca culture in southern Peru, the Moche civilization in northern Peru flourished between 200 and 800 AD (Fig. 1). Artefacts from the Moche Valley document a highly sophisticated Cu metallurgy during the late Moche period, including the first known appearance of arsenic bronze in South America^4^,^14^. Aside from the occurrence of pure Cu artefacts (tumi knives, pins, tweezers, belt ornaments) (Fig. 5), Au-Cu-Ag alloys were also found (crowns, headdresses, necklaces)^14^,^31^. Here, Cu was added to improve the mechanical properties and to produce the culturally required colour of a metal object^14^. The Sicán culture, following the Moche in northern Peru, reached the height of their copper production during the Middle and Late Period (~900–1350 AD), with copper smelting centred on Batán Grande in the Lambayeque valley, producing vast amounts of arsenic bronze^32^,^33^.

The Moche, Sicán, and later the Inca used two types of furnaces to smelt mainly Cu oxides (e.g. cuprite) or Cu-carbonates (e.g. malachite)^28^,^34^,^35^,^36^: (a) Pit furnaces dug into the ground or ceramic pots: Workers used simple tubes to blow on the fire they had built inside this furnace to reach temperatures capable of smelting ores; (b) Wind furnaces (huayras): These were built from clay, had an opening on top for charging ore and charcoal, a large hole in the front for retrieving the metal and initial lightning of the fire, and smaller holes around the circumference. As the wind blew over the small holes, it provided sufficient oxygen for the fire to burn hot enough for ore smelting^28^,^34^,^35^. The smelting of Cu sulphide ores was much more challenging. It included roasting the ores to obtain oxide ores and direct smelting the oxide ores with a CO producing fuel, or cosmelting sulphide with an oxide ore. Thus, sulphide ores were used much later compared to oxide and carbonate ores.

Partly overlapping with the Moche civilization, the Wari Empire in the north and the Tiwanaku Empire in the south became powerful during the Middle Horizon (~400–1000 AD) (Fig. 1). For both cultures there is convincing archaeological evidence for sophisticated Cu metallurgy. These cultures initiated a change from the utilization of Cu to the intentional use of bronze, which was heavily employed to manufacture needles, knives, and tupu pins^14^. Depending on the prevailing ore types, either arsenic bronze (northern Andes)^4^,^37^,^38^ or tin bronze was produced, becoming the most important alloy of the south-central Andes by the end of the Middle Horizon^38^,^39^,^40^. Of 208 metal artefacts excavated at Tiwanaku sites (including tupu pins and brooches, finger rings, knives, and needles)^41^ only a few consisted of pure Cu or arsenic bronze, whereas the majority were either tin bronze or copper-arsenic-nickel (Cu-As-Ni) alloys. This unique ternary bronze alloy was also deployed to produce I shaped architectural cramps utilized in setting the stone blocks in buildings and Tiwanaku pyramids (Fig. 5)^4^,^42^. The use of this Cu-As-Ni alloy and the tradition of architectural cramps were restricted to the Tiwanaku culture. Inca buildings rarely, if ever, utilized cramps^42^.

The manifold archaeological evidence for advanced Cu metallurgy during the late Moche and Tiwanaku/Wari time is corroborated by the maximum in ice core Cu EFs from 500 to 850 AD. Parallel with the decline of the Wari and Tiwanaku empires at the end of the Middle Horizon the Cu EFs in the ice core decreased to background levels and remained low during the following Late Intermediate period (LIP; ca. AD 1000–1450) (Fig. 4). This period is characterized by decentralized polities in Central South America and the emergence of the Sicán culture in northern Peru. There is well-documented archaeological evidence for strong metallurgical activities of these cultures, including smelting sites as well as precious and utilitarian Cu objects primarily made of arsenic bronze^14^,^33^. However, source regions of air pollution arriving at the Illimani site do not include the far north coast of Peru (see back trajectory analyses, Fig. 1), explaining the discrepancy between low ice core Cu EFs and archaeological findings.

### Cu maxima between the 15^th^ and 18^th^ century - Incas and Colonial times

Two maxima of the Cu EFs exceeding the background levels were detected during the period 1400–1500 AD and 1600–1750 AD. They both show lower enrichment and duration than the previous maxima. In the Inca empire (~1438–1532 AD) Cu-As alloys continued to be produced, but tin bronze was the most widely used alloy for the manufacturing of ornaments, implements, and weapons and became the imperial bronze^38^,^43^ and a symbol of the Inca domination^44^. The strength and superior casting properties of this alloy allowed for making tumis, chisels, and other cutting tools (Fig. 5). Metallurgical production was brought to a new level in the Andes during the Inca time by reorganizing the labour force to shift to large-scale production of Cu alloys^45^.

The Hispanic conquest of Peru and Bolivia started ~1532 AD. Throughout the following Colonial period Cu EFs exceeded the background level only during 1600–1750 AD, clearly surpassed by Cu emissions from the Early Horizon civilizations (700–50 BC) and the Middle Horizon cultures (500–850 AD). The lower Cu EF levels during the Inca and Colonial times are consistent with technological and cultural developments suggested by artefacts and documentary data. First, there was a strong change in the technology for smelting Cu, starting initially with smelting malachite in open fires and advancing finally to the use of slag-forming furnaces, which release less pollution. Second, during Colonial times, Cu was regarded as a “plebeian” metal because of its relative low value compared to Ag and Au. It was predominantly used as ballast for ships returning to Spain, and for cannons, bells, less valuable coins, and household utensils^46^ (Fig. 5). Additionally, due to scarcity of fuel in the Andes, these resources were mainly dedicated to smelt Ag ores. Cu ores were exported for smelting in Europe and the US, where plentiful mineral coal was available. Consequently, the importance of Cu smelting in the Andes diminished strongly during Colonial times^47^, which may also explain why ice core Cu EFs did not indicate significant anthropogenic emissions in the 19^th^ century.

### Modern Cu mining and smelting, air pollution control

Ice core Cu EFs started rising again from the beginning of the 20^th^ century, parallel with the world-wide growing use of Cu for electrical applications (Fig. 5), which boosted Cu production in South America. Illimani Cu EFs in the second half of that century are unprecedented in the context of the last 6500 years, implying severe air pollution related to the soaring large-scale open-cast Cu mining and enhanced smelting activities in Chile.

For most of the 20^th^ century Chile was the world’s leading Cu producer, accounting currently for about one third of the global Cu production. The largest mines in Chile are Chuquicamata and Escondida in Northern Chile, opened in 1882 and 1981, respectively. The Chuquicamata mine was the biggest pit mine in the world during the 1990s, producing ~500,000 t Cu per year^48^ and has only recently lost its foremost position at the expense of the new Escondida Cu mine, which produces ~750,000 t per year^49^. Mined Cu concentrate in Chile is processed in seven Cu smelters^50^, with the Chuquicamata smelter being one of the largest Cu smelters in the world and producing ~535,000 t Cu per year.

While Cu ore extraction in South America (dominated by Chilean production) is still growing, Cu EFs started decreasing from the end of the 1980s on (Fig. 6). The timing is in line with first implementations of air pollution control measures, changes in Cu production technology, and an intensified export of raw Cu concentrates. The Chagres smelter was the first plant effectively regulated for PM10 and SO_2_ emissions in 1985^51^. Emissions from the Chuquicamata smelter came under regulation in 1991, after an air quality monitoring network was established in the region in 1986. Wet gas electrostatic precipitators were incorporated into the gas handling systems to remove heavy metal dust. Furthermore, since 1990, most of the growth in Cu production is related to foreign-owned operations. Thus, Cu concentrate is exported for smelting elsewhere or hydrometallurgical processing is performed, which does not require smelting^51^. Until the 1980s, all Chilean smelters used conventional or oxygen-injection reverberatory furnaces. In 1986, the first flash furnace was installed at Chuquicamata allowing a reduction in pollution levels^52^.

### Importance of Cu versus Ag metallurgy

**Figure 2:**
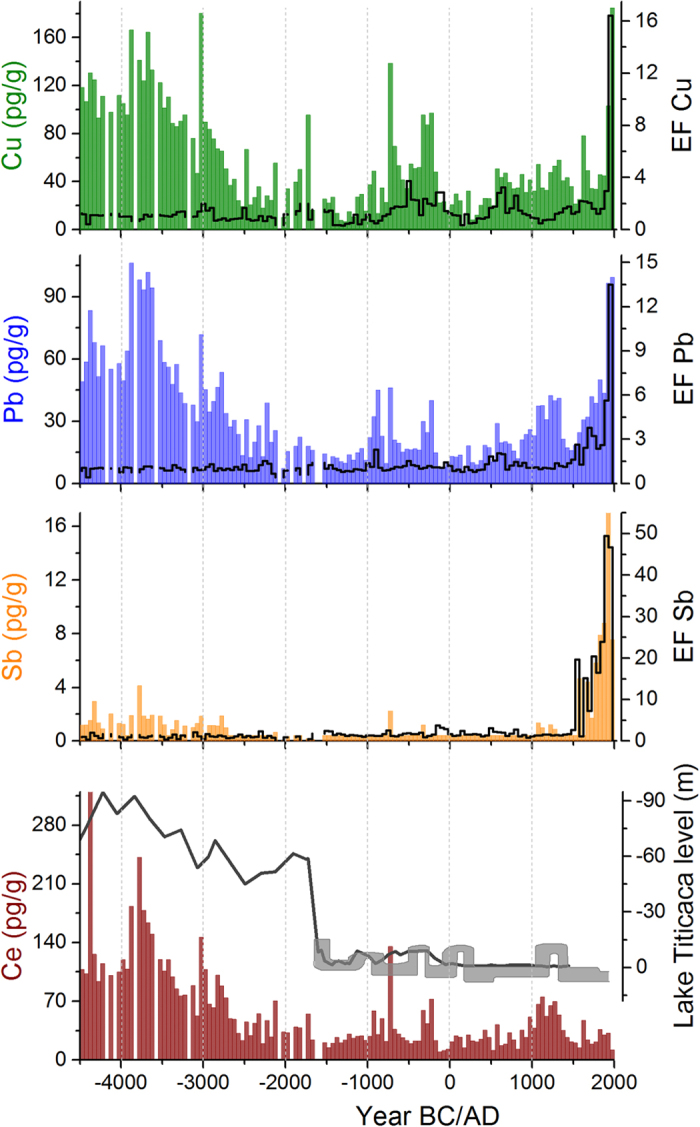
Ice core records of Cu, Pb, Sb, and Ce concentrations together with EFs, and lake levels of the Lake Titicaca for the period 4500 BC–2000 AD. Concentrations (bars) and EFs (black lines) are shown as 50-year medians. Pb data for the period 0–2000 AD are from ref. 21. Reconstructed lake levels of the Lake Titicaca are additionally presented in black^23^ and grey^60^ (inverse scale).

Throughout the 2700 years history of Cu metallurgy in South America, Cu objects primarily served to communicate social status, political power, and religious authority and awe^14^. Because of its colour and mechanical properties Cu formed the basis for Andean metallurgy. There is strong evidence from previous work on the Illimani ice core focussing on the past 2000 years^21^ and from lake sediments^20^,^53^ that extensive Ag metallurgy in South America only started around ~450 AD, somewhat later than the earliest archaeological evidence from Lake Titicaca^54^,^55^. Unlike Cu, Ag smelting emits only limited amounts of Ag into the atmosphere because of the low volatility of the pure metal and its compounds. Instead, Ag metallurgy can be traced by Pb emissions, since it is inextricably linked to smelting lead-rich ores, such as PbS. Our 6500-years Pb record (Figs 2 and 4) shows earliest increased Pb EFs above background from 500 to 750 AD, presenting evidence that the onset of extensive Cu smelting preceded that of widespread Ag metallurgy by about 1200 years. The dominance of Cu metallurgy holds true until Inca times^46^. In contrast, mining activities during the Colonial period were more oriented towards precious metals such as Ag^47^, used to produce objects representative of a high status and coins, whereas Cu production declined. Accordingly, Cu EFs exceed the background levels only during 1600–1750 AD, while Pb EFs remain well above background levels until the present (Fig. 4). The shift in mining-targeted metal during Colonial time is also distinctly documented in the concentration records of other ice core trace elements contained in Ag-bearing minerals, such as antimony (Sb, Fig. 2) or bismuth (Bi)^16^. Ag production in the Andes increased particularly after 1545 AD, when the legendary silver mountain Cerro Rico at Potosí was discovered. During this time, mined Ag was even shipped to China^56^. The final turnaround in favour of Cu metallurgy occurred in the 20^th^ century, related to the growing Cu demand for electrical industry and the opening of the Chilean mines, corroborated by highest Cu EFs. Thus, we conclude that the different scales of Cu and Pb EFs in the ice core represent the varying relevance of Cu and Ag metallurgy in South America, revealing that within the past 2700 years the high importance of Cu metallurgy was surpassed by that of Ag only during Colonial times.

### Comparison with other archives

**Figure 3:**
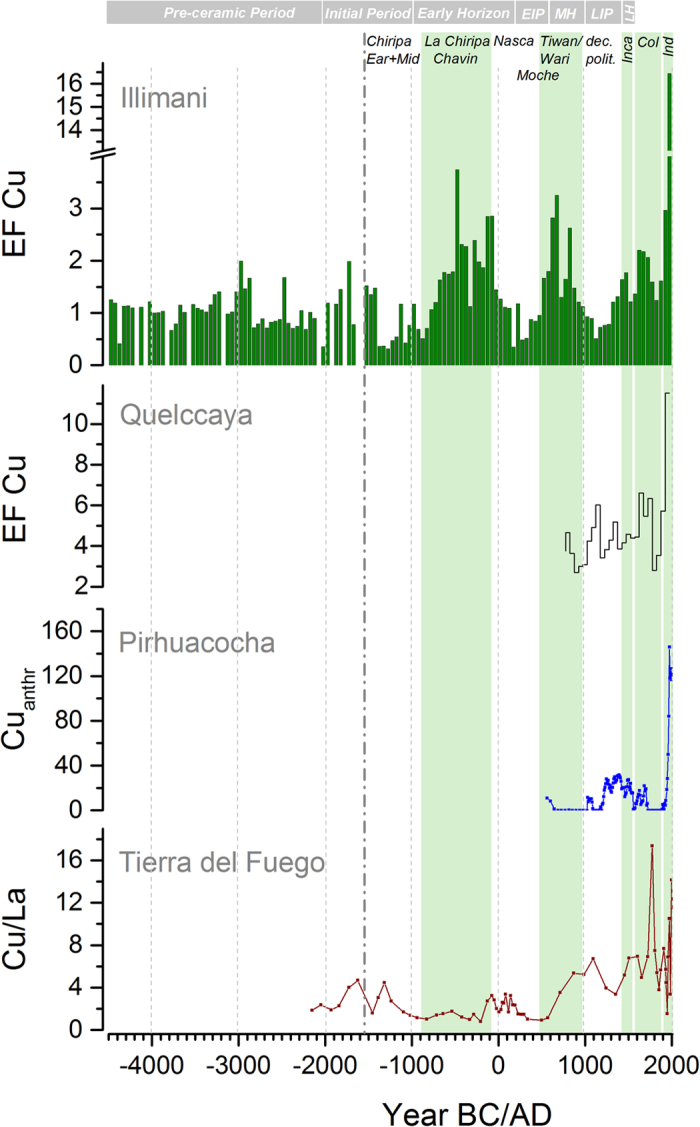
Illimani Cu EFs compared to Cu records from other natural archives in South America. Illimani Cu EF record (green, 50-year medians) together with Quelccaya ice core Cu EFs^16^ (grey, 50-yr medians), sediment core anthropogenic Cu from Laguna Pirhuacocha^17^ (blue, non-equidistant data), and peat bog Cu/La ratios from Tierra del Fuego^15^ (brown, non-equidistant data). Periods of generalized Andean archaeological history together with dominating cultures are indicated by green shaded areas.

**Figure 4:**
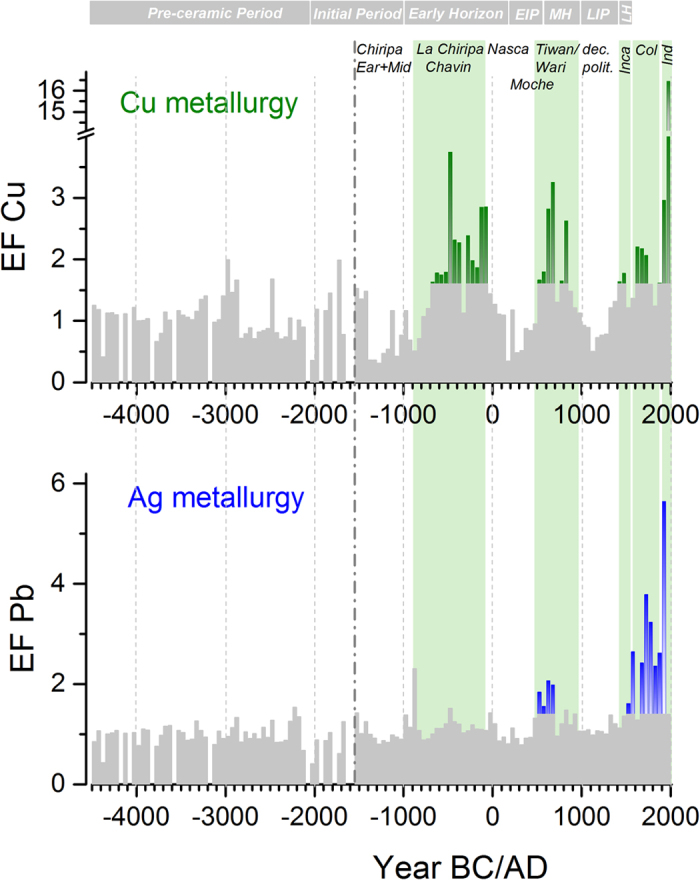
Ice core records of Cu and Ag metallurgy. Illimani Cu EFs (4500 BC–2000 AD) (green) and Pb EFs (4500 BC–1950 AD) (blue) exceeding in at least two consecutive data points (at least 100 years) the background (mean +2σ level of the period 4500–2000 BC) (grey) were considered to be anthropogenic Cu and Pb pollution from extensive Cu and Ag metallurgy, respectively. Pb EFs for the period 1950–2000 AD are not shown, since they represent emissions from leaded gasoline^21^. Periods of generalized Andean archaeological history together with dominating cultures are indicated by green shaded areas.

**Figure 5:**
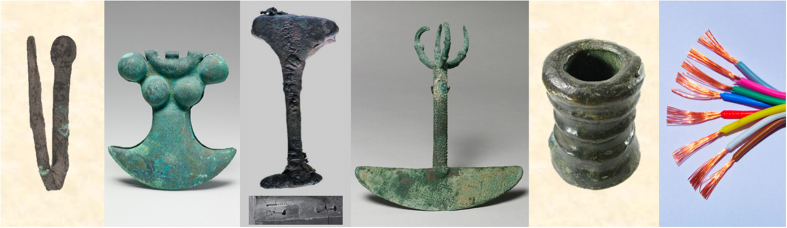
Cu artefacts documenting Cu metallurgy during periods with enhanced anthropogenic Cu emissions. From the left: (1) Chiripa Cu pin, bent, excavated by W. Bennett in 1934 at the Chiripa House 2 site (CH-H2^13^), occupied during the late Chiripa period (~600–100 BC)^12^ (Courtesy of the Division of Anthropology, American Museum of Natural History, #41.1/3895); (2) Moche belt ornament (Peru, 2^nd^–7^th^ century), Credit: The Metropolitan Museum of Art, www.metmuseum.org, bequest of Jane Costello Goldberg, from the Collection of Arnold I. Goldberg, 1986; (3) I shaped architectural cramp from the Puma Punka pyramid at Tiwanaku, Bolivia, composed of Cu-As-Ni bronze alloy^42^; (4) Inca tumi (ceremonial knife) made of tin bronze (Peru, 15^th^- early 16^th^ century), Credit: The Metropolitan Museum of Art, www.metmuseum.org, bequest of Jane Costello Goldberg, from the Collection of Arnold I. Goldberg, 1986, (5) Large bronze signal cannon, Spanish Colonial, 1600 s, Peru; Credit: Daniel Frank Sedwick, LLC, www.sedwickcoins.com; (6) modern Cu wires (Image: Anja Eichler).

**Figure 6:**
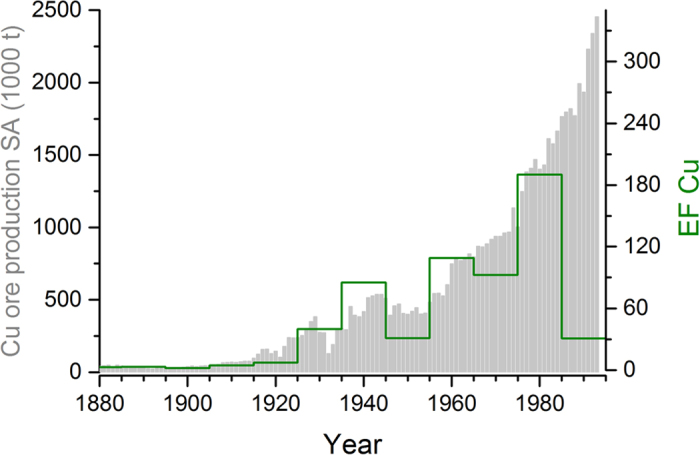
Ice core records of Cu EFs and Cu ore production in South America. Illimani Cu EFs (10 year means, green) and annual Cu ore production in South America^61^ (grey) are shown.

For the last 1200–1400 years, the time covered by the Cu records from the Peruvian Quelccaya ice core^16^ and the sediments of Lake Pirhuacocha^17^, there is good agreement with the general trends of the Illimani Cu EFs (Fig. 3). All records show unprecedented high Cu enrichments during the 20^th^ century due to Cu pollution from the massive Cu mining and smelting in Chile. The Cu EF decline at the end of the Colonial period is likewise depicted by all three records. The only exception is the LIP (1000–1450 AD), where the two Peruvian records suggest Cu pollution, whereas the Illimani Cu EFs decreased to background levels. The respective Pb records show a similar discrepancy. This is likely due to the slightly different catchment areas of the different archives, with the Peruvian records including contamination from the Middle and Late Sicán copper production at the far north coast of Peru. Furthermore, the uncertainty of the chronologies might also play a role. Two different age models provided for the dating of the Pirhuacocha core, for instance, differ by about 400 years during the LIP^17^.

To our knowledge the 4200-year peat bog Cu record from Tierra del Fuego is the only data set covering the entire time period of potential Cu metallurgy^15^. This record suggested earliest anthropogenic Cu pollution and the onset of pre-Columbian Cu metallurgy in South America as far back as ~2000 BC and shows a first prolonged non-dust Cu maximum during ~1700–1300 BC (see Fig. 3), which contradicts the findings from the Illimani ice core and the absence of archaeological evidence for sustained Cu use during this Initial Period. Other generally observed features such as highest values during the 20^th^ century from large scale Cu production are not seen in the peat bog record. Tierra del Fuego at the tip of South America is located in the Westerly circulation belt, 3,000 km south of the major centres of Cu metallurgy in the Central Andes, implying that the pollution signal is more diluted relative to the natural background. Due to the limited period covered by the peat bog record the variability in the natural background composition could not be taken into account, possibly explaining to some extent the discrepancy with the other records and the archaeological evidence.

## Conclusions

The 6500-year ice core Cu record from the Illimani glacier in the Bolivian Andes has yielded insight into the earliest stages of extensive prehistoric Cu metallurgy in South America and the timing and intensity of historical Cu smelting activities. Enrichment factors of Cu relative to the lithogenic element Ce were used to adjust for natural Cu deposition with mineral dust, which was particularly pronounced in the dry period before ~2800 BC. Strongly fluctuating Cu EFs from 4500 to 2000 BC with no metal-producing society settled in the Andes at that time highlight the importance of considering variability in the mineral dust composition to discriminate natural input from metallurgy-related emissions. This is especially crucial for identifying the likely weak signal of prehistoric stages of extractive metallurgy from geochemical analyses of natural archives. We detected earliest anthropogenic Cu pollution during the Early Horizon period ~700–50 BC. Thus, despite little archaeological evidence for Cu exploitation at this early time, we propose an onset of intensified Cu smelting in South America during the central Andean Chiripa and Chavin cultures about 2700 years ago. Our data do not confirm a potentially earlier onset of extractive Cu metallurgy during the Initial Period, as suggested by the 4200-year peat bog Cu record from Tierra del Fuego.

In contrast to the onset of Cu smelting, our Cu EF signatures otherwise match the archaeologically known periods of increased metal production during the last two millennia, and the different relative importance of Cu and Ag metallurgy for pre- and post-conquest societies is clearly visible in the different scales of Cu and Pb EFs. The onset of extensive Cu smelting preceded that of widespread Ag metallurgy in South America by about 1200 years. Cu emissions from the earliest Chavin/Chiripa civilizations and the Late Moche/Tiwanaku/Wari cultures were found to surpass those of the Inca and Colonial period. This can be related to changes in furnace technologies and a declining importance of Cu at the expense of an increasing exploitation of precious metals such as Ag during the Colonial era, as illustrated by the relative enhancement of Pb EFs compared to Cu EFs since the 16^th^ century. In agreement with records from two other natural archives in South America, highest Cu EFs occurred during the second half of the 20^th^ century, in line with severe pollution primarily from open-cast Cu mining and Cu metallurgy in Chile. In addition, the impact of air pollution control measures during the last few decades is well documented in the Illimani archive, serving as an example of the effect that such technological changes can have on the environment and the corresponding record.

## Methods

### Ice core archive

In 1999, the 138.7 m long ice core investigated in the present study was retrieved from Nevado Illimani (16°37′S, 67°64′W, 6300 m asl) in a joint expedition of the Swiss Paul Scherrer Institut (PSI) and the French Institut de Recherche pour le Développement (IRD)^18^. Low borehole temperatures (−7 °C at 10 m depth, −8.9 °C at 65 m, and −8.4 °C near bedrock) together with very few melt features indicate well-preserved records of past air pollution levels. The mean annual net accumulation of 0.58 m weq. allows for highly time-resolved chemical records^18^. The timescale for the ice core was derived by a combination of different dating methods. These included annual layer counting (ALC) and ^210^Pb dating for the upper 75 m, the use of known reference horizons from six volcanic eruptions (AD 1258, 1815, 1883, 1963, 1982, 1991) and from the tritium maximum in AD 1964, together with ^14^C dating for the lowest 18 m^18^,^57^. A continuous age-depth relationship was established by fitting a two-parameter glacier flow model through the reference horizons and the ^14^C dates^57^. The upper 134 m of the ice core used in this study cover the period BC 4500–AD 1996. The dating uncertainty of the ALC until about AD 1800 is estimated to ≤2 years in the vicinity of the volcanic time markers and ≤5 years between the volcanic horizons. The errors associated with the dating model are ≤20 years in the period AD 0–1800, and ≤110 years in the period BC 4500–0.

### Ice core sampling and analyses of Ce, Cu, La, Nd, Pb, and Sb

In the −20 °C cold room at PSI individual core sections (length 60–75 cm, diameter 7.8 cm) were cut for trace element analysis. Inner sections with a diameter of 2.2 cm × 2.2 cm were cut out of the cores using a modified stainless-steel band saw with a Teflon coated saw guide and tabletop. With the continuous ice melting device, a final decontamination is directly performed with the melting head, separating potentially contaminated melt water of the surface from that of the innermost part of the core^58^,^59^. For selected core sections, continuous ice melting was not possible because of poor ice core quality. Here, discrete samples were rinsed with ultrapure water (18 MΩ cm quality) in a class 100 clean bench and transferred into pre-cleaned high-density polyethylene containers. Subsequently, these samples were acidified with ultrapure HNO_3_ to yield a final concentration of 0.2 N HNO_3_.

The analysis of trace element concentrations was performed with continuous ice melting inductively coupled plasma–sector field–mass spectrometry (CIM-ICP-SF-MS) using Element 1 (Thermo Finnigan MAT) for the deepest ice core parts (34 to 134 m) and Element 2 (Thermo Scientific) for the upper 34 m applying standard procedures^21^,^58^,^59^. The melt water of the inner drain of the melting head was acidified to a final concentration of 0.2 N HNO_3_ and injected into the mass spectrometer using a microflow self-aspirating PFA nebulizer of an APEX Q sample introduction system (Elemental Scientific Inc.). Discrete samples were introduced to the APEX system with an autosampler (221 XL, Gilson Inc. and ASX-260, Cetac, respectively). For the present study Ce, Cu, La, Nd, Pb, and Sb were analyzed using an external calibration with liquid calibration standards. Calibration correlation coefficients were generally higher than 0.999. Prior to the analysis of each ice core section procedural blank concentrations were determined by analyzing an identically prepared artificial ice bar of ~10 cm length^58^. Every ice core section was then corrected by its individual procedural blank. Average procedural blank levels for Ce, Cu, La, Nd, Pb, and Sb were 0.15, 10, 0.1, 0.08, 3, and 0.3 pg/g, respectively.

### Data availability

Data will be available at the NOAA (National Oceanic and Atmospheric Administration) data center for paleoclimate (ice core sites) after acceptance of the paper: http://www.ncdc.noaa.gov/data-access/paleoclimatology-data/datasets/ice-core.

## Additional Information

**How to cite this article:** Eichler, A. *et al*. Ice-core evidence of earliest extensive copper metallurgy in the Andes 2700 years ago. *Sci. Rep.* **7**, 41855; doi: 10.1038/srep41855 (2017).

**Publisher's note:** Springer Nature remains neutral with regard to jurisdictional claims in published maps and institutional affiliations.

## Supplementary Material

Supplementary Information
