# Supplementary material for: Ice-core evidence of earliest extensive copper metallurgy in the Andes 2700 years ago
Source: Sci Rep. 2017 Jan 31;7:41855. doi: 10.1038/srep41855 (PMC5282569; doi:10.1038/srep41855)
Supplement: Supplementary Information [file srep41855-s1.pdf]

SUPPLEMENTARY INFORMATION

Ice-core evidence of earliest extensive copper metallurgy in the Andes 2700 years ago

A. Eichler [1,2]*, G. Gramlich[1,2,3], T. Kellerhals[1,2], L. Tobler[1,2], Th. Rehren[4], and M. Schwikowski[1,2,3]

[1] Paul Scherrer Institut, CH-5232 Villigen PSI, Switzerland

[2] Oeschger Centre for Climate Change Research, University of Bern, CH-3012 Bern, Switzerland

[3] Department for Chemistry and Biochemistry, University of Bern, Freiestrasse 3, CH-3012 Bern, Switzerland

[4] UCL Institute of Archaeology, 31-34 Gordon Square, London WC1H 0PY, UK

*Corresponding author:

e-mail: anja.eichler@psi.ch, phone: +41 56 310 2077, fax: +41 56 310 4435

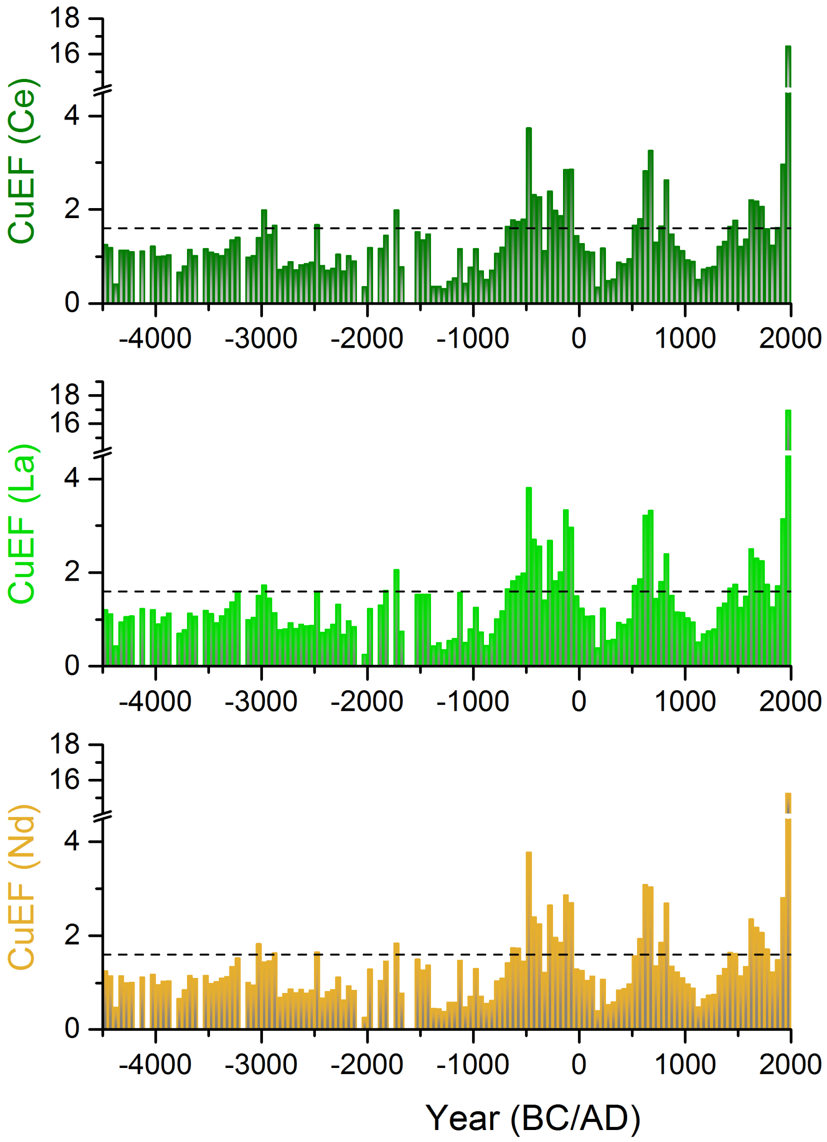

Supplementary Figure S1: Comparison of CuEF records deduced using different lithogenic elements (Ce-dark green, La-light green, Nd-yellow). CuEFs are shown as 50 yr medians. The dashed lines represent the background level from mineral dust input of EF=1.6 (mean+2σ level of the period 4500-2000 BC). Only CuEFs exceeding the background level in at least two consecutive data points are considered to be anthropogenic Cu pollution from extensive Cu metallurgy.
